# Comparative Phylogenomics Reveals Conserved Protoilludene Synthase Neighbourhoods and Unevenly Distributed Biosynthetic Gene Cluster Families in Armillaria

**DOI:** 10.3390/jof12090677

**Published:** 2026-09-10

**Authors:** Huijun Su, Ming Luo, Yuhuan Miao, Xiaohua Li, Dahui Liu, Wei Su

**Affiliations:** 1 School of Pharmacy, Hubei University of Chinese Medicine, Wuhan 430065, China; 2 Hubei Shizhen Laboratory, Wuhan 430065, China

**Keywords:** Armillaria, biosynthetic gene cluster, comparative genomics, fungal specialised metabolism, gene cluster family, genome mining, protoilludene synthase, terpene

## Abstract

Fungal biosynthetic gene clusters (BGCs) offer a genomic entry point for natural-product discovery, but predicted regions alone neither establish pathway activity nor identify metabolites. Armillaria is a useful model because chemically rich, forest-associated species span pathogenic and saprotrophic lifestyles. We analysed nine assemblies in a role-aware design: seven independent taxa with high completeness assessed using Benchmarking Universal Single-Copy Orthologs (BUSCO), an *A. mellea* BUSCO duplication-sensitivity sample and an *A. solidipes* intraspecific strain control for *A. ostoyae*. The analysis resolved a terpene-rich repertoire containing a shared but edge-robust gene cluster family (GCF) component alongside unevenly distributed candidate families. Targeted neighbourhood analysis recovered protoilludene synthase (PRO1) orthologs in all assemblies (92.2–100.0% amino-acid identity); the core neighbourhood was retained across the seven primary taxa, whereas flanking tailoring-associated genes varied. Notably, the *A. gallica* PRO1 ortholog lay 23,614 bp outside the nearest antiSMASH region. Thus, whole-region prediction can obscure pathway-centred conservation in basidiomycetes. The resulting framework prioritises reproducible candidates while reserving chemical identity, expression and ecological function for direct validation.

## 1. Introduction

Fungal specialised metabolites mediate competition, host interactions and responses to environmental stress, and they remain an important source of pharmacological and biotechnological leads [1]. Many of the enzymes, transporters and regulators required for their biosynthesis are encoded in biosynthetic gene clusters (BGCs). Genome mining can therefore reveal biosynthetic potential that is not apparent from routine culture experiments. However, the presence of a predicted BGC is not evidence that the locus is expressed, that its encoded enzymes are active or that a specific metabolite is produced. Developmental state, environmental conditions and chromatin regulation can silence or reshape secondary metabolism [1,2]. A useful comparative analysis must consequently distinguish genomic prediction from biochemical confirmation.

Species of Armillaria are globally distributed forest-associated basidiomycetes that include destructive root pathogens, wood decomposers and fungi with complex ecological associations [3,4]. This ecological breadth, together with a melleolide-rich chemistry, makes the genus a useful model for examining BGC evolution in Basidiomycota. Their species boundaries and evolutionary relationships have been investigated using multilocus and genome-scale data [5,6]. Armillaria chemistry is dominated by structurally diverse sesquiterpene aryl esters, including melleolides, which show antimicrobial, cytotoxic and other bioactivities [7]. Biochemical and genetic studies have identified protoilludene synthase, a polyketide synthase, cytochrome P450 monooxygenases, a GMC oxidoreductase and chlorination steps involved in melleolide biosynthesis [8,9,10,11,12,13,14]. However, how the corresponding pathway-centred neighbourhoods vary across a phylogenetically controlled genus-wide panel—including conservation of scaffold-forming genes and turnover of tailoring-associated genes—has not been systematically resolved.

Raw BGC-region counts provide only a limited comparative view. Fungal clusters can change through duplication, loss, rearrangement, horizontal transfer and divergence in gene content [15], whereas differences in assembly continuity and annotation can alter predicted region boundaries. Gene cluster family (GCF) methods group related regions and enable prevalence to be compared across genomes [16,17,18]. When combined with a phylogenomic framework, explicit sampling roles and sensitivity analyses, GCF occupancy can separate predictions that are stable across a panel from those that are restricted to fewer sampled taxa. Reference-cluster comparisons provide an additional evidence layer, but a database match remains a computational similarity result and the absence of a match does not demonstrate novelty.

Here, we used nine Armillaria genome assemblies in a role-aware comparative design. Seven independent taxa constituted the primary-core panel for prevalence inference; *A. mellea* was included as a BUSCO duplication-sensitivity sample, whereas *A. solidipes* was retained as an intraspecific strain control for *A. ostoyae*. We tested the working hypothesis that a conserved protoilludene synthase-centred scaffold is embedded within more variable flanking tailoring-gene neighbourhoods, and that region-boundary calling can obscure this pathway-centred conservation. We integrated genome completeness, single-copy-marker phylogenomics, antiSMASH prediction, BiG-SCAPE GCF inference, edge sensitivity, pairwise GCF similarity, KCB evidence and targeted neighbourhood analysis.

## 2. Methods

### 2.1. Taxon Panel and Assembly Quality Control

Nine Armillaria genome assemblies were obtained from NCBI GenBank (Table 1). Seven assemblies representing independent taxa constituted the primary-core set: *A. mexicana* (GCA_052426525.1), *A. fumosa* (GCA_030407015.1), *A. nabsnona* (GCA_030407065.1), *A. ostoyae* (GCA_964034915.1), *A. altimontana* (GCA_022818075.1), *A. cepistipes* (GCA_900157415.1), and *A. gallica* (GCA_002307695.1). *A. mellea* (GCA_030407055.1) was retained as the BUSCO duplication-sensitivity sample because its 16.9% duplicated BUSCOs make it informative for assessing duplication-associated sensitivity, rather than as an eighth independent prevalence taxon. *A. solidipes* (GCA_002307675.1) was retained as an intraspecific strain control for *A. ostoyae* because the two share a near-identical taxonomic affiliation; it was not treated as an additional independent taxon.

Both genome- and protein-mode BUSCO assessments were run. Reported BUSCO values quantify annotation completeness: protein BUSCO completeness was assessed with BUSCO v5.8.0 against fungi_odb10 (protein mode, *n* = 758) on AGAT-selected longest-isoform protein sequences [19,20]. Repetitive elements were masked with RepeatModeler v2.0.8 [21] and RepeatMasker v4.2.3.

### 2.2. Gene Annotation

Gene models were predicted with BRAKER3 v3.0.3 [22] in protein-only mode using 37,931 UniProtKB/Swiss-Prot fungal proteins as evidence, with Armillaria sequences excluded to prevent self-training bias. BRAKER3 was executed in Singularity. AGAT v1.1.0 was used to select the longest isoform per gene from the AUGUSTUS-derived GFF3 output; the selected protein FASTA was used for protein-mode BUSCO. Gene-level feature counts therefore represent AGAT longest-isoform gene features rather than raw BRAKER3 transcript counts (Table 1; Table S5).

### 2.3. BGC Prediction

BGC regions were predicted with antiSMASH v8.0.4 [23] using softmasked FASTA and AGAT longest-isoform, AUGUSTUS-derived GFF3 annotations supplied simultaneously through —genefinding-gff3 <longest.gff3> and —genefinding-tool none, with —taxon fungi and —minlength 200. The primary detection runs did not enable —cb-general or other optional general ClusterBlast modules; KCB and SubClusterBlast were run subsequently as described below. The —minlength setting is an antiSMASH record-length filter rather than a preprocessing step. A region was classified as contig-edge only when antiSMASH reported contig_edge=True; no distance threshold was applied.

KnownClusterBlast (KCB) analysis against MIBiG v4.0 was performed for eight genomes through antiSMASH —reuse-results —cb-knownclusters. For *A. cepistipes*, reuse was incompatible with the original detection JSON format; KCB was obtained in a fresh run using identical frozen detection parameters, and detection-level consistency was verified for all 56 regions with zero product, coordinate or contig-edge mismatches. A region was KCB-supported when knownclusterblast.total_hits > 0, without additional similarity, coverage or ranking thresholds. SubClusterBlast was subsequently run with —cb-subclusters for the same eight reuse-compatible genomes and through the consistency-verified fresh *A. cepistipes* run. The MIBiG ClusterCompare module was not executed because the required local database files were absent; functional reference evidence therefore derives from KCB and SubClusterBlast as explicitly reported.

### 2.4. GCF Clustering

Region-level BGCs were clustered with BiG-SCAPE v2.0.3 [24] using —classify class, —alignment-mode glocal, —gcf-cutoffs 0.2,0.3,0.4 and Pfam 35.0. No additional —is-fungal flag was supplied; clustering used the stated v2.0.3 options on antiSMASH fungal-mode region GBKs. The primary cutoff was 0.30, with 0.20 and 0.40 treated as sensitivity analyses; all nine assemblies contributed to one GCF universe. Default BiG-SCAPE mix distance weights were retained for class-specific comparisons. Pairwise Jaccard similarity was calculated from the 7 × 74 primary-core binary presence/absence matrix at cutoff 0.30, excluding singletons, the mellea-only GCF and both non-primary-core assemblies.

### 2.5. Proto-Core-Associated CDS

CDS features, region boundaries and proto-core intervals were parsed from main GBK files in one parent-contig coordinate system. A CDS was proto-core-associated when its coordinates overlapped an antiSMASH proto-core interval by at least 1 bp. CDS with gene_kind annotations containing ‘biosynthetic’, including both ‘biosynthetic’ and ‘biosynthetic-additional’, were classified as biosynthetic gene_kind CDS.

### 2.6. Phylogenomic Analysis

Strict single-copy BUSCO markers present in all seven primary-core taxa were identified: 661 of 758 fungi_odb10 markers were retained. Sequences were aligned with MAFFT v7.526 [25] using —auto and concatenated into 458,074 amino acid positions. Maximum-likelihood inference used IQ-TREE v3.1.2 [26] with 661 marker partitions, ModelFinder Plus (-m MFP) [27] for per-partition best-fit model selection, 1000 ultrafast bootstraps, 1000 SH-aLRT replicates and —seed 42. The primary-extended tree used 535 markers and 382,513 positions. Trees were midpoint-rooted for visualisation only.

### 2.7. Targeted Protoilludene Synthase and Neighbourhood Analysis

Experimentally characterised A. gallica protoilludene synthase sequences (P0DL13.2 and AGR34199.1) were used as references [8,28]. Predicted longest-isoform proteomes were searched with DIAMOND v2.2.4 in sensitive mode (E ≤ 1 × 10^−20^; query and subject coverage ≥ 70%), and reciprocal-best hits were retained as ortholog candidates. Selected region proteins were compared with the fungal UniProtKB/Swiss-Prot database using DIAMOND (E ≤ 1 × 10^−5^) and scanned against Pfam 35.0 with HMMER v3.3.2 using curated gathering thresholds (—cut_ga). Neighbourhood homology was analysed with clinker v0.0.32. Orthogroup-level conservation was summarised across the seven primary taxa, and adjacent-neighbourhood protein links with ≥50% identity were used for the static R visualisation. For A. gallica, where the ortholog fell outside an antiSMASH region, the ortholog-centred flanking interval was retained. Track orientation follows the deposited assemblies; differences in arrow direction were not interpreted as evolutionary inversions because contig and scaffold orientation can be arbitrary.

## 3. Results

### 3.1. High Annotation Completeness Supports Comparative BGC Profiling

Because BGC calls depend on the underlying gene set, we first assessed annotation completeness across the comparative panel. Protein-mode BUSCO completeness ranged from 96.7% to 98.7%, and AGAT longest-isoform gene models ranged from 11,382 to 15,940 per assembly (Table 1; Table S5). These consistently high values provide a suitable basis for comparing predicted BGC repertoires, while the separate *A. mellea* duplication-sensitivity sample retains potential duplication effects outside the primary prevalence panel.

### 3.2. Genome-Wide Profiling Reveals a Terpene-Dominated BGC Landscape

**Figure 1:**
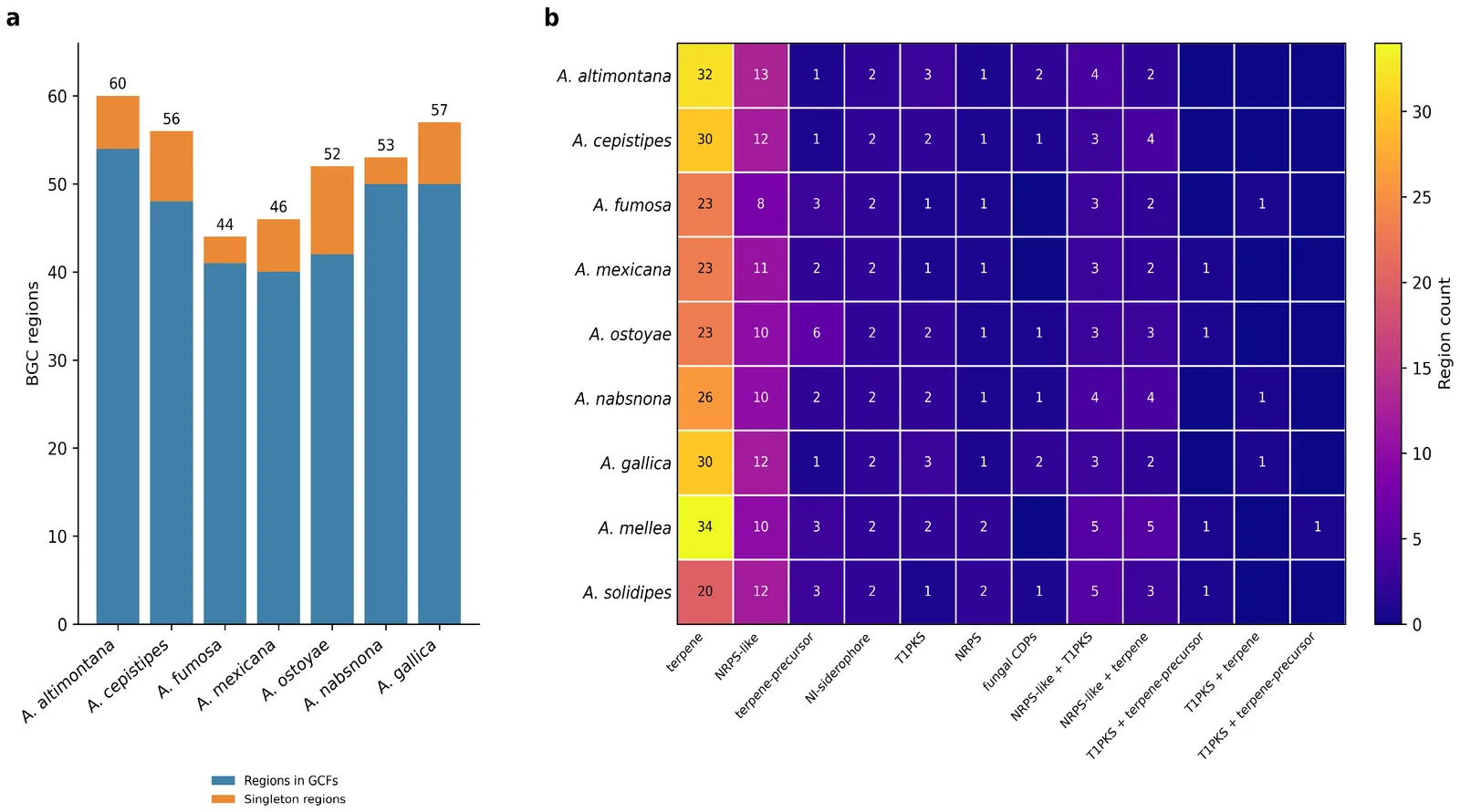
BGC region accounting and product-combination distribution. (**a**) Primary-panel BGC regions shown as stacked counts of regions in GCFs and singleton regions; total bar height equals BGC regions. (**b**) Heatmap of mutually exclusive normalised product combinations across all nine assemblies. Each region is assigned to one of 12 combinations; colour and printed integers show region counts.

To define the genomic landscape before inferring sharedness, we standardised antiSMASH product-label order and examined region-level composition. The 483 BGC regions spanned 12 unique product combinations (Table S1; Figure 1). Terpene was the largest single-product class (241/483; 49.9%), and 276 regions carried a terpene label when hybrid combinations were included; NRPS-like regions were the next largest single-product class (98/483; 20.3%). Hybrid assignments occurred in 68 regions. Only 6 of these 68 hybrid regions were contig-edge calls (8.8%), compared with 23 of 415 non-hybrid regions (5.5%); this descriptive contrast does not indicate that the hybrid status is an assembly-edge artefact. The pattern therefore identifies terpene capacity as the dominant prediction-level signal while retaining hybrid calls as separate, auditable product combinations.

### 3.3. A Shared Biosynthetic Core Is Embedded Within Uneven GCF Occupancy

**Figure 2:**
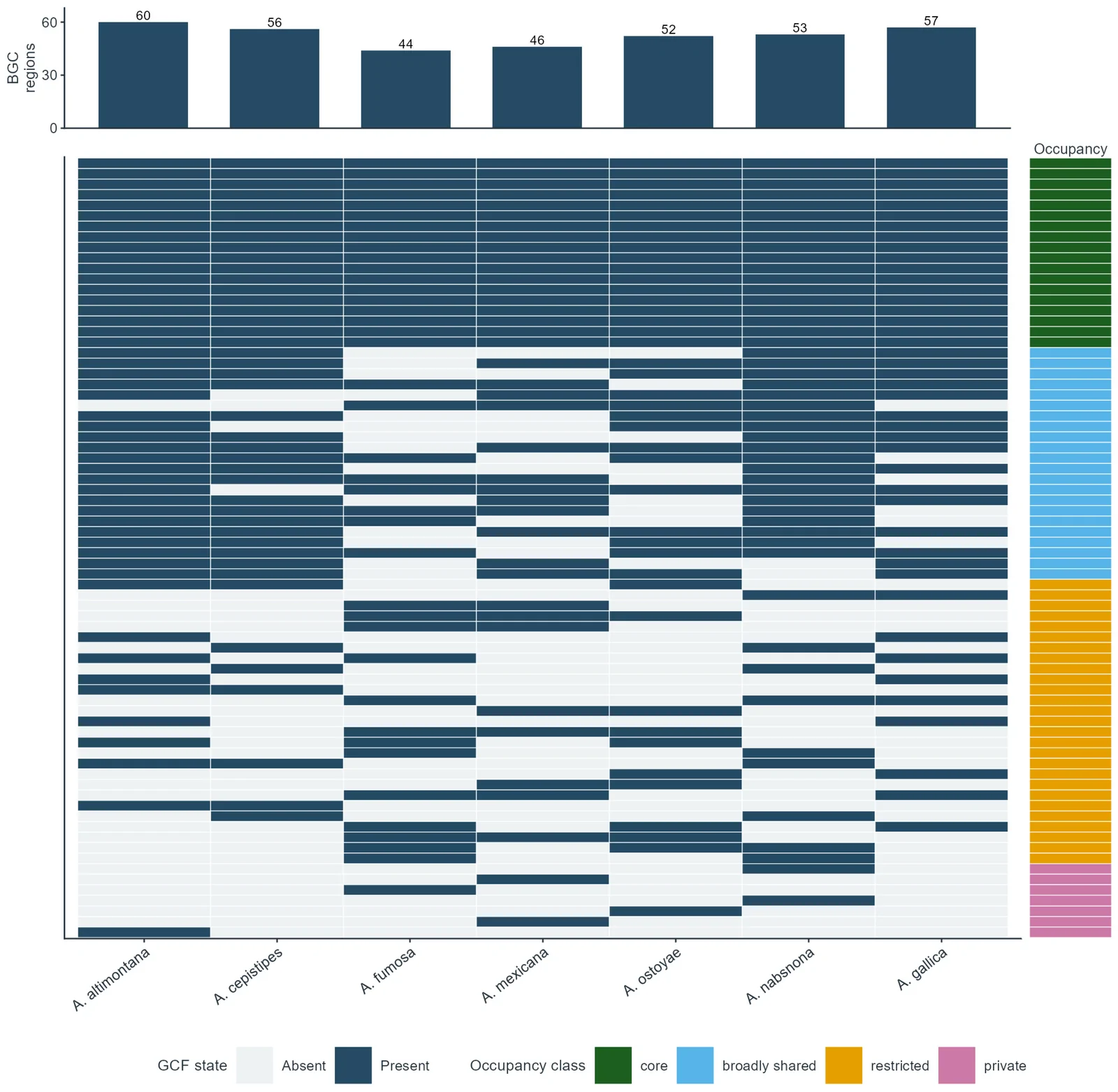
Primary-panel GCF occupancy matrix. Binary presence/absence of 74 GCFs detected in at least one of seven primary taxa at the BiG-SCAPE 0.30 cutoff. GCFs are grouped by mutually exclusive occupancy class, columns follow the Figure 1 phylogenomic order, and the top bars show total BGC-region counts per taxon. The occupancy strip uses the same discrete colour scheme as Figure 4. *A. mellea*, *A. solidipes*, 59 singleton regions and the mellea-only GCF are excluded.

To separate simple region abundance from recurrent gene-cluster architecture, we clustered all region calls into GCFs and evaluated their occupancy in the seven independent taxa. At the primary cutoff of 0.30, 424 regions formed 75 GCFs and 59 remained singletons. Of the 74 GCFs detected in the primary panel, 18 were core, 22 broadly shared, 27 restricted and 7 private (Table 2; Figure 2). Thus, a shared component coexists with a larger set of unevenly distributed GCFs; occupancy is a comparative detection pattern and does not by itself establish expression, copy number or chemical equivalence.

### 3.4. Cutoff, Edge and Strain Controls Preserve the Main Occupancy Pattern

To test whether the main pattern was driven by clustering threshold, assembly-edge calls or the designated controls, we performed cutoff, edge-filter and role-aware sensitivity analyses. Cutoffs of 0.20 and 0.40 yielded 78 and 70 GCFs, respectively, with stable occupancy profiles (Table 2). Removal of 12 contig-edge regions from the primary panel changed the classification of only three GCFs, and 16 of 18 core GCFs retained 7/7 occupancy. Extension to the *A. mellea* primary-extended set added one mellea-only GCF. *A. ostoyae* and its *A. solidipes* intraspecific strain control shared 34 of 42 and 43 clustered GCFs, respectively, as well as 730 single-copy BUSCO markers. Together, these analyses show that the central occupancy signal is robust to the tested technical and sampling controls.

### 3.5. GCF Similarity Only Partly Follows Phylogenomic Proximity

**Figure 3:**
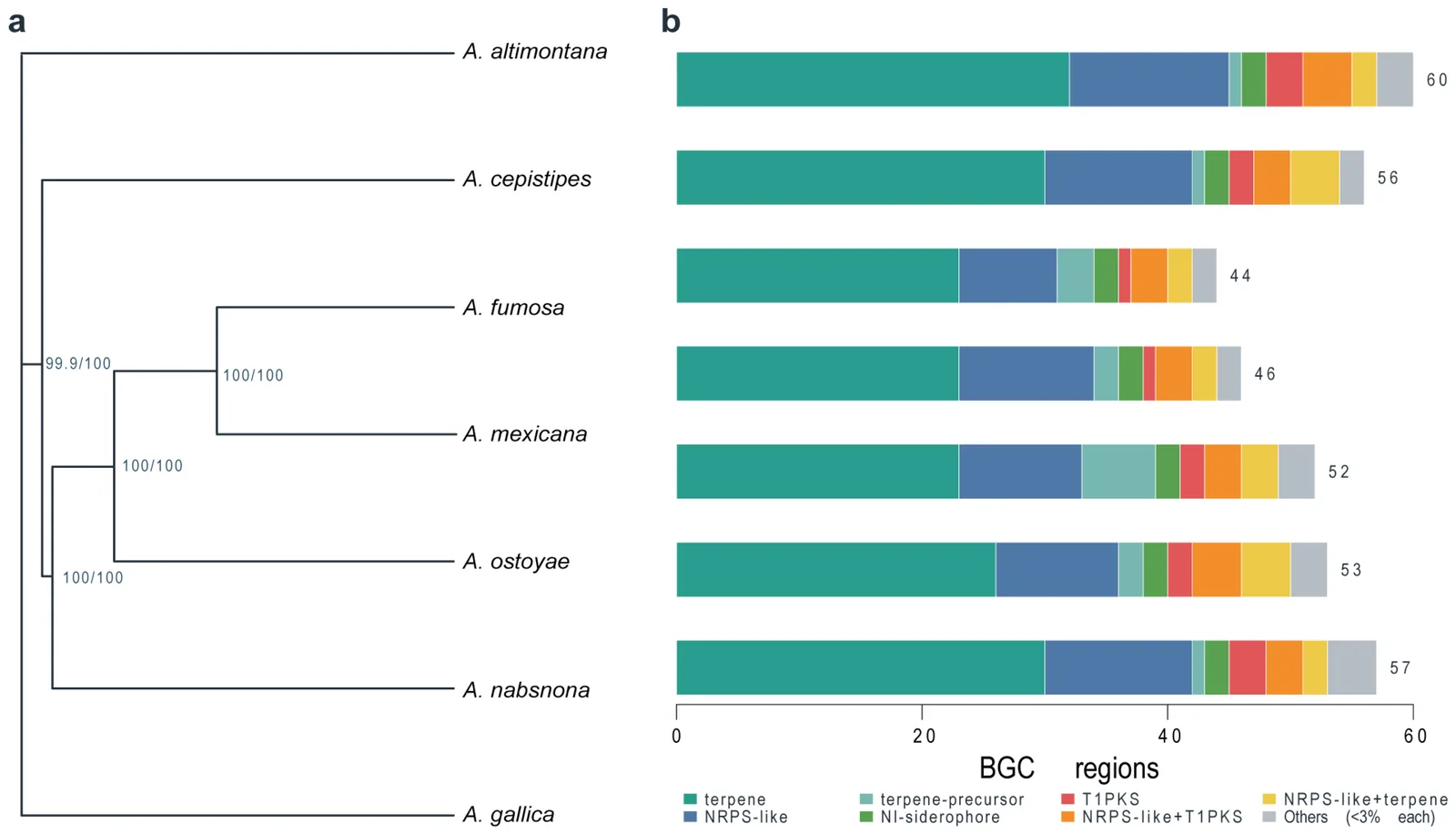
Phylogenomically ordered Armillaria BGC landscape. (**a**) Maximum-likelihood tree of seven primary taxa inferred from 661 single-copy BUSCO protein markers (458,074 amino acid positions). Internal-node labels are SH-aLRT/ultrafast-bootstrap support; all nodes received 100% ultrafast bootstrap and ≥99.9% SH-aLRT support. The midpoint root is for visualisation only. (**b**) Mutually exclusive product-combination counts for each primary taxon; stacked-bar length equals its total predicted BGC regions. Product combinations contributing <3% of primary-panel regions are combined as Others in this panel only.

To place BGC sharing in an evolutionary context, we inferred a phylogeny from 661 single-copy BUSCO markers and compared the corresponding GCF profiles. All internal nodes received 100% ultrafast-bootstrap and ≥99.9% SH-aLRT support (Figure 3). Pairwise GCF Jaccard similarity ranged from 0.407 to 0.769 (mean 0.545), with the highest similarity between *A. altimontana* and *A. cepistipes* and the lowest between *A. cepistipes* and *A. fumosa*. This partial concordance provides phylogenetic context for BGC sharing, but it does not imply that each predicted family follows the species tree.

### 3.6. Most GCFs Lack Matches to Characterised Reference Clusters

To distinguish recurrence from similarity to characterised chemistry, we assessed KnownClusterBlast evidence and proto-core content for each region. KCB hits occurred in 53 of 483 regions and covered 12 of 75 GCFs; 63 GCFs had no KCB hit (Tables S2 and S3). Notably, 15 of the 18 core GCFs lacked KCB support, showing that broad occupancy does not guarantee representation in the characterised reference space. All 75 GCFs contained at least one proto-core-associated CDS. Of 6525 region CDS, 713 were proto-core-associated and 1589 carried a gene_kind annotation containing ‘biosynthetic’, including both biosynthetic and biosynthetic-additional assignments (Table S4). SubClusterBlast returned no hits for any of the 483 regions. Because the antiSMASH v8.0.4 installation contained 126 shipped reference subclusters that were all bacterial, this zero-hit outcome is interpreted as a database-coverage limitation, not evidence that fungal subcluster homologs are absent.

### 3.7. Orthogonal Evidence Prioritises Edge-Stable Terpene Candidates

**Figure 4:**
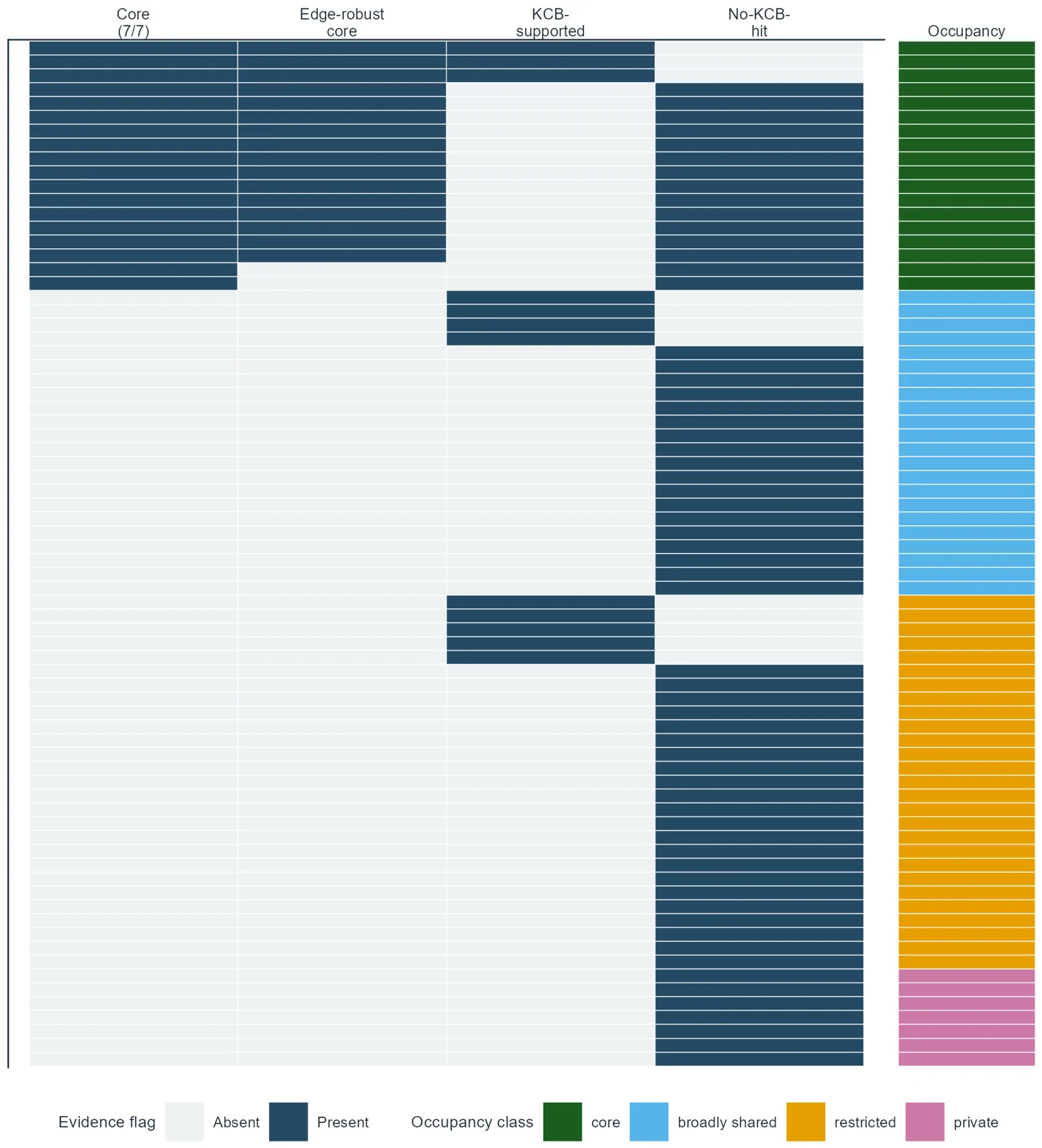
Evidence matrix for primary-panel GCFs. The 74 primary-panel GCFs are shown against four overlapping binary flags: core (7/7), edge-robust core, KCB-supported and no-KCB-hit. The high-contrast right strip denotes occupancy class. KCB-supported and no-KCB-hit are mutually exclusive; 12 and 62 GCFs, respectively, meet these definitions.

To prioritise loci without conflating prevalence and functional assignment, we integrated occupancy, edge sensitivity and reference evidence in Figure 4. Among 74 primary-panel GCFs, 18 were core, 16 were edge-robust core, 12 were KCB-supported and 62 had no KCB hit. The matrix makes the divergence in evidence explicit: 3 core GCFs were KCB-supported, whereas 15 core GCFs remained no-hit; both edge-sensitive core GCFs were also no-hit. Two edge-stable no-hit terpene GCFs were selected as sequence-level candidates (Table S9). GCF 0.3|terpene|46 contained one *A. ostoyae* region and one *A. solidipes* region and remained private to one primary taxon after edge filtering; it is distinct from the classical PRO1-containing *A. ostoyae* locus. GCF 0.3|terpene|49 was present in *A. gallica*, *A. ostoyae* and *A. solidipes* and contained several sesquiterpene synthase-like proteins. These loci are computational targets only: similarity annotations do not establish metabolite identity, expression, toxicity, virulence or ecological function.

### 3.8. Conservation and Structural Variation in Protoilludene Synthase Neighbourhoods

**Figure 5:**
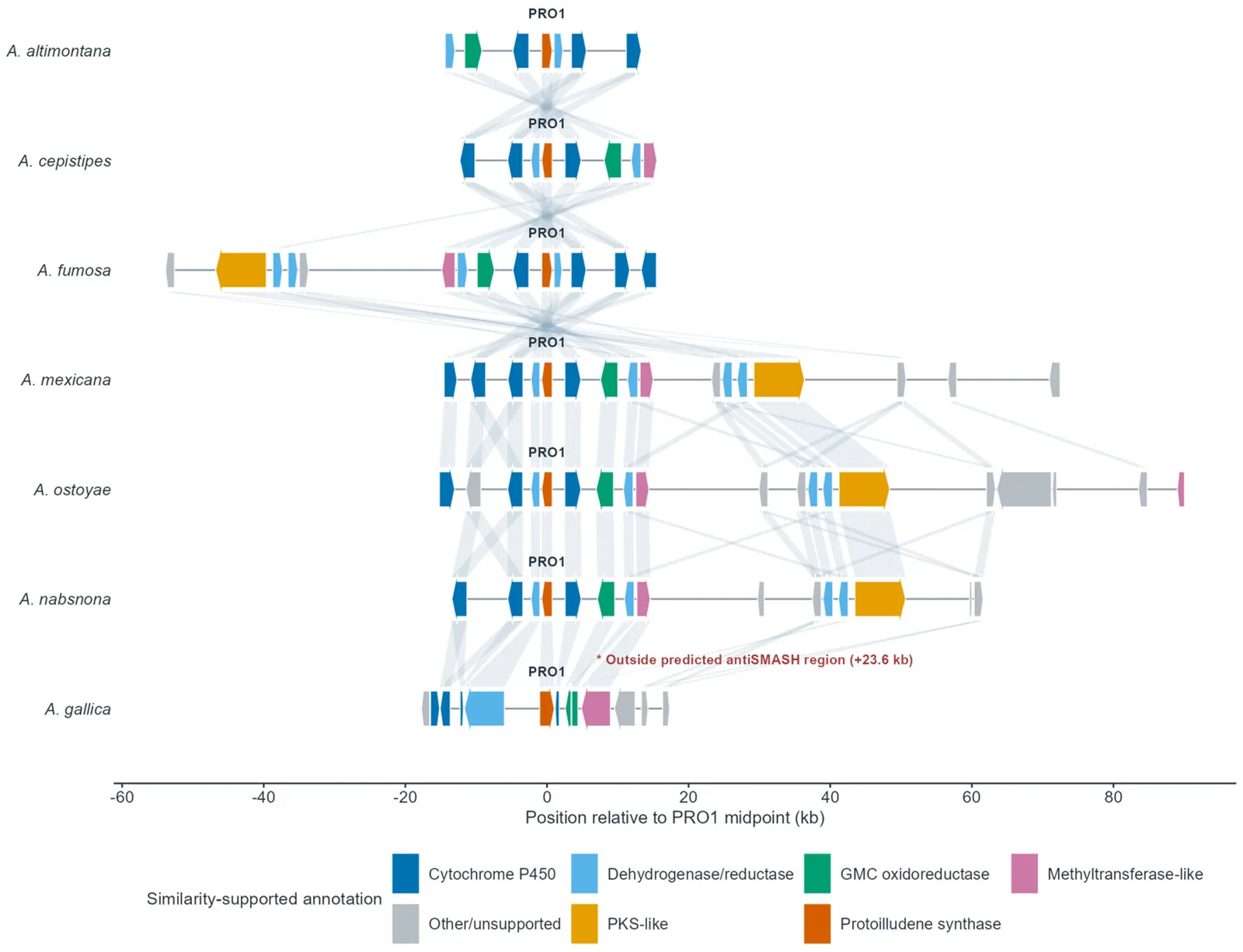
Conservation and structural variation in PRO1-centred neighbourhoods across seven primary Armillaria taxa. Gene arrows are positioned relative to the PRO1 midpoint and coloured by similarity-supported functional category. Translucent ribbons connect adjacent-track proteins with ≥50% amino acid identity. PRO1, a dehydrogenase/reductase-like orthogroup and a GMC oxidoreductase-like orthogroup are conserved, whereas flanking genes vary. The *A. gallica* PRO1 locus is shown as an ortholog-centred window and lies 23.6 kb outside the nearest predicted antiSMASH region (red asterisk label). Assembly track orientation is retained, and directional differences must not be interpreted as evolutionary inversions.

Because whole-region clustering can mask a conserved pathway backbone, we independently examined neighbourhoods centred on protoilludene synthase (PRO1). Reciprocal-best-hit searches recovered a PRO1 ortholog in all nine assemblies, with 92.2–100.0% amino acid identity, 99.7% query coverage and 100% subject coverage relative to the selected reference (Table S7). In the seven primary taxa, the PRO1 orthogroup, a dehydrogenase/reductase-like orthogroup and a GMC oxidoreductase-like orthogroup were present in all taxa; a methyltransferase-like orthogroup occurred in six. Cytochrome P450-like genes were also represented across all seven neighbourhoods, with three to four similarity-supported CDS per taxon, whereas wider flanking complements varied (Figure 5; Table S10). Although *A. fumosa* and *A. mexicana* are sister taxa in the genome-scale tree, their PRO1-centred profiles differ in the presence and arrangement of flanking similarity-supported genes. This observation is consistent with local gain, loss, duplication or rearrangement after species divergence, but the present similarity-based analysis does not resolve the evolutionary mechanism. The high PRO1 amino acid identity across all assemblies supports conservation of the scaffold-forming enzyme; a dedicated PRO1 gene tree and formal tests of selection will require a broader curated sequence set and are an important next step. antiSMASH and BiG-SCAPE partitioned the eight region-contained orthologs among GCF65 (terpene), GCF222 (T1PKS + terpene) and GCF135 (T1PKS + terpene + terpene-precursor), despite conservation of the PRO1-centred neighbourhood. Crucially, the *A. gallica* PRO1 ortholog lay 23,614 bp upstream of the nearest predicted antiSMASH region (GCF222). This single, audited boundary miss demonstrates why targeted neighbourhood analysis is complementary to automated region calls; it does not quantify an overall antiSMASH error rate.

## 4. Discussion

This analysis resolves a central feature of predicted specialised metabolism across the sampled Armillaria genomes: a stable shared component is embedded within a larger unevenly distributed repertoire. Eighteen GCFs occurred in all seven independent primary taxa, and sixteen remained core after removal of contig-edge regions. In parallel, 56 GCFs were broadly shared, restricted or private within the primary panel. The result is more informative than a comparison of region counts alone because occupancy was calculated at the taxon level, interpreted in a phylogenomic context and tested against assembly-edge and control sensitivities.

Terpene regions dominated the predicted repertoire: 241 of 483 regions were single-product terpene predictions, and 276 regions carried a terpene label when hybrid combinations were included. This signal is consistent with the exceptional chemical prominence of Armillaria sesquiterpene aryl esters, but it should not be equated with 276 distinct pathways or products. More than 70 melleolide-type compounds had been reported by 2024, and metabolomic analysis of *A. ostoyae* resolved 22 melleolide-associated molecular families containing 277 features, including dimers and fatty-acid-substituted congeners [7,12]. Comparative profiling of pathogenic *A. mellea* and saprotrophic D. ectypa further showed that related armillarioid fungi can share the melleolide scaffold while differing in detected congeners [13]. Together with characterised protoilludene synthase, polyketide synthase, P450, GMC oxidoreductase and chlorination chemistry [8,9,10,11,14], the data are consistent with a modular or combinatorial organisation in which a conserved scaffold-forming capacity is paired with variable tailoring reactions [29].

The targeted neighbourhood comparison gives this model a gene-level basis. PRO1 and two enzyme-associated orthogroups were conserved in all seven primary taxa, whereas methyltransferase-like, PKS-like and other flanking components varied. Importantly, the same pathway-centred neighbourhood was split among three GCFs, and one *A. gallica* locus lay outside the nearest antiSMASH region. This does not invalidate whole-region clustering; it shows that region callers and GCF algorithms describe a different analytical scale from an ortholog-centred pathway comparison. Product-label combinations, flanking-gene content and predicted boundaries can separate regions even when a central biosynthetic neighbourhood is conserved.

The explicit control design reduced two common sources of overinterpretation. First, *A. mellea* had 16.9% duplicated BUSCOs and 14 contig-edge regions, yet its addition produced only one control-only GCF at the main cutoff and did not disrupt the topology among primary taxa. Second, 730 shared single-copy BUSCO markers and 34 shared GCFs between *A. ostoyae* and *A. solidipes* supported their treatment as closely related control assemblies rather than independent taxonomic replication. These controls do not eliminate annotation or assembly effects, but they make the inferential unit explicit.

KCB added an orthogonal evidence axis. Twelve GCFs included at least one region with similarity to a reference BGC and are natural priorities when the aim is to connect candidates to characterised chemistry. The 63 no-hit GCFs remain useful discovery targets, but the no-hit status means only that antiSMASH reported no KCB match in MIBiG v4.0 under the recorded run; it does not demonstrate novelty. This limited correspondence is biologically plausible because curated BGC reference space has historically been uneven: MIBiG v3.0 listed 415 Ascomycota entries but only 23 Basidiomycota entries [30]. The absence of SubClusterBlast matches is even less informative here because the installed reference collection was bacterial-only. GCF46 and GCF49 illustrate how the no-hit status can be combined with edge stability, restricted occupancy and gene-level similarity annotation to define experimentally tractable targets without labelling them as toxins or pathogenicity determinants. GCF46 is an edge-stable, KCB-no-hit terpene family represented by one *A. ostoyae* region and one *A. solidipes* region; it includes an STS-like protein but is distinct from the classical PRO1-containing *A. ostoyae* locus. GCF49 is an edge-stable, KCB-no-hit terpene family detected in *A. gallica*, A. ostoyae and *A. solidipes* and contains several sesquiterpene-synthase-like proteins. These module-level annotations prioritise hypotheses for expression, heterologous reconstruction and metabolite profiling; they do not establish products or pathway membership (Tables S2, S9 and S10).

Several limitations define the scope of the conclusions. BGC prediction is sensitive to assembly continuity, gene annotation, region-boundary algorithms and representation of basidiomycete cluster architectures in reference databases. MIBiG ClusterCompare could not be executed because the required local database files were absent, and the available SubClusterBlast database lacked fungal references. Clinker relationships and orthogroups are similarity-based; they do not by themselves establish enzyme function, pathway membership or evolutionary rearrangement. In particular, deposited contig orientation prevents directional differences in Figure 5 from being interpreted as inversions. Biologically relevant genes may occur outside compact predicted regions, as demonstrated by the *A. gallica* PRO1 locus, and a detected region does not establish transcription, metabolite production or ecological function.

These limitations point directly to a validation workflow. Representative GCFs can first be ranked using independent attributes such as edge-robust prevalence, reference-cluster support, product-combination prediction, proto-core gene content and ortholog-centred neighbourhood conservation. The current data do not establish which species produces the highest melleolide or sesquiterpene aryl ester titres. Nevertheless, for targeted discovery, *A. ostoyae* and its *A. solidipes* control are practical comparative candidates for GCF46 because both contain the family, whereas *A. gallica*, *A. ostoyae* and *A. solidipes* are the appropriate comparative set for GCF49. For GCF46, matched vegetative-mycelium, rhizomorph and wood- or host-contact conditions in A. ostoyae and *A. solidipes* would test whether its STS-like gene is transcriptionally responsive before targeted disruption and LC-MS/MS analysis. For GCF49, matched mycelial, rhizomorph and, where obtainable, fruiting-body samples from *A. gallica*, *A. ostoyae* and *A. solidipes* would provide a comparative expression screen before heterologous reconstruction of the STS-like module. These are proposed validation conditions, not inferred functions; targeted gene disruption or heterologous expression and LC-MS/MS molecular networking linked to purified compounds are required before any connection to host interaction, toxicity or virulence can be proposed.

## 5. Conclusions

A role-aware comparison of nine Armillaria assemblies identified 483 predicted BGC regions and organised 424 clustered regions into 75 GCFs. The sampled repertoire was terpene-rich and contained both an edge-robust shared component and a larger unevenly distributed component. Targeted analysis revealed a conserved PRO1-centred neighbourhood across the primary taxa despite variable flanking genes, multiple whole-region GCF assignments and an antiSMASH boundary miss in *A. gallica*. Reference-cluster similarity covered only 12 GCFs, while edge-stable no-hit terpene GCFs provided focused candidates for experimental characterisation. These results establish a phylogenetically controlled and scale-aware foundation for prioritising Armillaria specialised-metabolism loci while leaving pathway activity, chemical products and ecological roles to direct validation.

## Figures and Tables

**Table 1:** Assembly quality, gene annotation and BGC accounting across nine Armillaria assemblies.

Taxon	Accession	Role	BUSCO_C (%)	BUSCO_D (%)	Genes	BGC Regions	Regions in GCFs	Singleton Regions	Contig-Edge Regions	KCB-Hit Regions
*A. altimontana*	GCA_022818075.1	Primary core	98.5	1.6	15,709	60	54	6	4	8
*A. cepistipes*	GCA_900157415.1	Primary core	98.7	2.5	15,848	56	48	8	2	5
*A. fumosa*	GCA_030407015.1	Primary core	98.4	1.1	12,910	44	41	3	1	5
*A. gallica*	GCA_002307695.1	Primary core	98.4	2.8	15,527	57	50	7	3	7
*A. mellea*	GCA_030407055.1	BUSCO duplication-sensitivity sample	96.7	16.9	15,940	65	54	11	14	6
*A. mexicana*	GCA_052426525.1	Primary core	97.4	2.9	11,382	46	40	6	1	4
*A. nabsnona*	GCA_030407065.1	Primary core	98.7	1.6	14,482	53	50	3	1	7
*A. ostoyae*	GCA_964034915.1	Primary core	97.9	1.6	14,525	52	42	10	0	5
*A. solidipes*	GCA_002307675.1	Intraspecific strain control	98.7	1.6	14,914	50	45	5	3	6

Notes: BUSCO completeness was assessed in protein mode (BUSCO v5.8.0) on AGAT-selected longest-isoform protein sequences against fungi_odb10 (*n* = 758). C, complete BUSCOs; D, complete and duplicated BUSCOs. Full C/S/D/F/M values are presented in Table S5.

**Table 2:** GCF occupancy sensitivity across BiG-SCAPE cutoffs.

Occupancy Class	c0.20	c0.30 (Primary)	c0.40	Edge-Filtered c0.30
Core (7/7)	11	18	21	16
broadly shared (4–6)	24	22	22	23
Restricted (2–3)	33	27	21	28
Private (1/7)	8	7	5	7
Mellea-only	2	1	1	1
PC-present GCFs	76	74	69	
Total GCFs	78	75	70	
Singleton regions	110	59	40	

Notes: c0.30 is the primary cutoff. The edge-filtered column removes 12 contig-edge primary-core regions before recomputing occupancy. KCB evidence is reported separately in Figure 4 and Tables S2 and S3.

## Data Availability

The genome assemblies analysed in this study are publicly available from NCBI under the accessions listed in Table 1. The processed data that support the findings of this study are available from the corresponding authors upon reasonable request.

## Supplementary Materials

The following supporting information can be downloaded at https://www.mdpi.com/article/10.3390/jof12090677/s1, Table S1: Catalogue of 483 antiSMASH regions; Table S2: Catalogue of 75 GCFs with occupancy and KCB flags; Table S3: Region-level KnownClusterBlast evidence; Table S4a: All 6525 CDS records from predicted regions; Table S4b: Per-assembly reconciliation of CDS evidence; Table S4c: The 713 proto-core-associated CDS records; Table S5: BUSCO, phylogenomic and strain-control summary; Table S6: Software and reproducibility manifest; Table S7a: Verified protoilludene synthase reference metadata; Table S7b: Audited protoilludene ortholog and locus assignments; Table S8: SubClusterBlast evidence for 483 unique regions; Table S9: Similarity annotations for GCF46 and GCF49 candidate genes; Table S10: Orthogroups in seven primary PRO1-centred neighborhoods.
